# An Annotated Draft Genome for the Andean Bear, *Tremarctos ornatus*

**DOI:** 10.1093/jhered/esab021

**Published:** 2021-04-21

**Authors:** Nedda F Saremi, Jonas Oppenheimer, Christopher Vollmers, Brendan O’Connell, Shard A Milne, Ashley Byrne, Li Yu, Oliver A Ryder, Richard E Green, Beth Shapiro

**Affiliations:** 1 Department of Biomolecular Engineering and Bioinformatics, University of California Santa Cruz, Santa Cruz, CA; 2 Department of Medical and Molecular Genetics, Oregon Health & Science University, Portland, OR; 3 Department of Ecology and Evolutionary Biology, University of California Santa Cruz, Santa Cruz, CA; 4 Department of Molecular, Cellular, Developmental Biology, University of California Santa Cruz, Santa Cruz, CA; 5 State Key Laboratory for Conservation and Utilization of Bio-Resource in Yunnan, School of Life Sciences, Yunnan University, Kunming, China; 6 San Diego Zoo Global, Beckman Center, Escondido, CA; 7 Howard Hughes Medical Institute, University of California Santa Cruz, Santa Cruz, CA

**Keywords:** Oxford nanopore, CHiCago, Andean bear, spectacled bear, *Tremarctos ornatus*

## Abstract

The Andean bear is the only extant member of the Tremarctine subfamily and the only extant ursid species to inhabit South America. Here, we present an annotated de novo assembly of a nuclear genome from a captive-born female Andean bear, Mischief, generated using a combination of short and long DNA and RNA reads. Our final assembly has a length of 2.23 Gb, and a scaffold N50 of 21.12 Mb, contig N50 of 23.5 kb, and BUSCO score of 88%. The Andean bear genome will be a useful resource for exploring the complex phylogenetic history of extinct and extant bear species and for future population genetics studies of Andean bears.

Bears of the family Ursidae are a geographically widespread lineage of large mammals that are important to cultures around the world, from providing sustenance to inspiring stories and mythologies. Eight ursid species persist to the present day. Molecular and comparative morphological approaches have been used to infer their phylogenetic relationships and evolutionary histories (McLellan and Reiner 1994; McKenna and Bell 1997; Yu et al. 2007; Kutschera et al. 2014; Kumar et al. 2017; Cahill et al. 2018). The subfamily Ursinae includes brown bears, *Ursus arctos*; polar bears, *U. maritimus*; American black bears *U. americanus*, Asiatic black bears *U. thibetanus;* sloth bears, *U. ursinus*, and sun bears *U. malayarius*. Ursinae is sister to the monophyletic subfamily Tremarctinae, which includes *Tremarctos ornatus*, the Andean bear or spectacled bear. Outgroup to these subfamilies is the monophyletic Aliuropodinae, which includes the giant panda *Ailuropoda melanoleuca*. Together, species within Ursidae represent an enormous range of physiologies, dietary and behavioral strategies, and ecological adaptations. Comparative analyses of their genomes promise to improve our understanding of the genomic underpinnings of these traits.

Here, we present an annotated draft assembly of *Tremarctos ornatus*, commonly known as the Andean bear. The Andean bear is the only extant member of the Tremarctinae subfamily and the only extant bear in South America (Kurtén 1967). The presumed ancestor of *T. ornatus*, *T. floridanus*, is known only from North America, where it became extinct during the Holocene (Trajano and Ferrarezzi 1995). The oldest *T. ornatus* fossil was discovered in a cave in the Peruvian Andes and dates to around 6000 years ago (Stucchi et al. 2009). The absence of older *T. ornatus* from the fossil records of either North or South America suggests that the extant Andean bear lineage diverged from *T. floridanus* during the early Holocene (Soibelzon et al. 2005), although the timing of its dispersal into South America remains unknown. Today, Andean bears are found mainly along the Andes Mountains of northern and western South America, where they occupy habitats ranging from coastal deserts to high elevation grasslands and forests, often in small populations (Kattan et al. 2004; García-Rangel 2012). Andean bears are currently listed as “vulnerable” across this region by the IUCN, due to a combination of hunting pressure and increasing habitat fragmentation driven by human land use changes (Velez-Liendo X and García-Rangel 2016).

A high-quality de novo assembly of an Andean bear genome enables the application of a range of new molecular approaches in the study of this species and of other bears. Andean bears are the natural outgroup to the much better studied ursine bears and a de novo Andean bear genome will improve comparative analyses within that group. An Andean bear genome is also a crucial resource for reconstructing the evolutionary history within Tremarctinae, which, during the Pleistocene, also included the much larger and carnivorous short-faced bears, *Arctodus* and *Arctotherium* (Soibelzon and Schubert 2011). While well-preserved remains from both species exist (Mitchell et al. 2016), a closely related reference genome is key to accurately mapping the short and damaged fragments of DNA that are recovered from fossils (Shapiro and Hofreiter 2014). Finally, a reference Andean bear genome will facilitate the molecular characterization of wild populations of Andean bears that have thus far been limited to microsatellite or mitochondrial DNA analysis (Ruiz-Garcia 2003; Viteri and Waits 2009; Cueva et al. 2018).

To generate and annotate a de novo Andean bear genome, we use a combination of short-read (Illumina) and long-read (Oxford Nanopore Technologies, ONT) sequencing technologies to generate both DNA and RNAseq data. Our assembly is of Mischief, a captive-born Andean bear, and uses data generated from archived fibroblast cells. Our final genome has a length of 2.23 Gb across 12 402 scaffolds, with a scaffold N50 of 21.12 Mb, BUSCO score of 88%, and QV score of 34.36, making it of similar to or better quality than other *Ursidae* genome assemblies in terms of completeness and contiguity (Miller et al. 2012; Taylor et al. 2018; Fan et al. 2019; Srivastava et al. 2019), and provides an important resource for future research.

## Methods

### Biological Materials

We obtained fibroblast cells (passage 4) established by the Biodiversity Banking group at San Diego Zoo Global and archived in the Frozen Zoo® (Lab# 11593). The fibroblast cell line was derived from a skin biopsy taken from a captive-born female Andean bear named Mischief (GAN: MIG12-29960943 studbook number 513) that was housed at and owned by Phoenix Zoo.

### Nucleic Acid Library Preparation

#### Illumina Shotgun Data

We extracted DNA from fibroblast cells using the Qiagen DNeasy Blood & Tissue kit following the manufacturer’s protocol for non-nucleated erythrocytes. Following extraction, we concentrated the DNA via EtOH precipitation and then resuspended the pellet in 50 μL of TE buffer at 55 °C for 2 h. We then sheared the DNA using ultrasonication with a Covaris instrument (Covaris, Inc. Woburn, MA), targeting ~330 bp fragments, and made one single-indexed Illumina library following (Meyer and Kircher 2010). Paired-end sequencing of the library was performed at the Vincent J. Coates Genomics Sequencing Laboratory at UC Berkeley on an Illumina HiSeq 2500 (2 × 100 cycles) (Bentley et al. 2008).

#### CHiCago Data

We generated a CHiCago library using the fibroblast cells from Mischief using a previously described method (Putnam et al. 2016). We sent the library for sequencing at the UC San Diego Institute for Genomic Medicine Genomics Center on one lane of Illumina HiSeq 2500 (2 × 125 cycles).

#### Oxford Nanopore Data

To obtain high molecular weight genomic DNA for long-read sequencing, we performed a DNA extraction from 750 μL the fibroblast cells using the Qiagen Blood and Cell Culture Mini Kit, following manufacturer’s instructions until the spooling step. We concentrated the DNA via centrifugation via EtOH precipitation, and then resuspended the pellet in 50 μL of TE buffer on a shaker at 22 °C overnight. We quantified DNA with a Qubit 2.0 dsDNA HS kit, and verified the DNA size distribution with a pulse-field gel using a 0.75% agarose TAE gel, run at 75V for 16 h, with the preset 5–150 kb program on the Pippin Pulse power supply, and estimated the DNA fragments to average roughly 23 kb in length.

We performed a ligation sequencing run (Oxford Nanopore, SQK-LSK108). For the Rapid library preparation, we used an input of 1000 ng of DNA, as recommended by the manufacturer. The DNA was end-repaired and ligated to sequencing adapters using the SQK-LSK108 kit according to manufacturer’s instructions. We quantified the sequencing libraries using a Qubit prior to sequencing. The sequencing library was run on a MinION instrument with an R9 flow cell using the NC 48 h sequencing protocol. We base-called fast5 raw reads generated from the sequencing run using Albacore v2.0.2 (Oxford Nanopore proprietary).

#### Short Read RNAseq Data (Illumina)

We made a cDNA library from fibroblast cells using the NEBNext Poly(A) mRNA magnetic isolation protocol according to manufacturer’s instructions. We checked the library size distribution using an Agilent 2100 Bioanalyzer, and sequenced the library on one-half lane of Illumina HiSeq 2000 (2 × 75 cycles) at the Vincent J Coates Genomic Sequencing Laboratory.

#### Long Read RNAseq Data (ONT)

To obtain RNA from fibroblast cells we performed RNA extraction using TRIzol reagent (Thermofisher). RNA was resuspended in RNAse-free water and further purified using the RNeasy Kit (Qiagen) following manufacturers recommendations. RNA was then run on a Nanodrop to verify quantity and purity of the RNA. cDNA was generated using the Smartseq2 method (Picelli et al. 2014). Two microliters of total RNA was reverse transcribed using Smartscribe Reverse transcriptase (Clonetech) in a 10 μL reaction volume and reverse transcribed at 42 °C for 60 min, 70 °C for 15 min. The resulting cDNA was treated with 1:10 dilutions of RNAseA (Thermofisher) and Lambda Exonuclease (NEB) for 30 min at 37 °C. cDNA was then amplified using KAPA Hifi Readymix 2X (KAPA Biosystems) and incubated at 95 °C for 3 min, followed by 15 cycles of (98 °C for 20 s, 67 °C for 15 s, 72 °C for 4 min) and a final extension at 72 °C for 5 min. The amplified cDNA was eluted in 30 μL of DNA suspension buffer. cDNA was run on 2% agarose gel to verify quality and also checked for quantity using Qubit. The cDNA was then prepared for library prep using the 1D2 Sequencing Kit SQK-LSK308 protocol following the manufacturer’s instructions with the exception of the end-repair step. The incubation time was extended for 30 min during the end repair instead of the recommended 15 min. The sequencing library was run on an R9.5 flow cell using the NC 48 h sequencing protocol. We base-called fast5 raw reads using Albacore v2.0.2 (Oxford Nanopore proprietary).

### Sequencing and Genome Assembly

#### Nuclear Genome Assembly

A list of all programs and versions used throughout the assembly process is available in Table 1. We removed adapters from the Illumina shotgun library with SeqPrep2 (https://github.com/jeizenga/SeqPrep2), using the default parameters except that we increased the quality score cutoff to 15 (-q 15) and reduced the minimum length of trimmed reads to 25 bp (-L 25). We used Trimmomatic v0.33 (Bolger et al. 2014) to 1) remove remaining IS3 adapters using a seed mismatch of 2 and a simple clip threshold reduced to 5 for the shorter adapter sequence; 2) end trim using minimum qualities of 2 for leading ends or 5 for trailing ends of reads; 3) window quality trim, using a window size of 4 and a minimum quality of 15; and 4) remove reads shorter than 50 bp. We then used this processed shotgun data to assemble a de novo genome with the Meraculous-2D Genome Assembler v2.2.4 (Chapman et al. 2011), with diploid mode set to 1 and a kmer size of 51. We scaffolded the Meraculous genome using HiRise v2.1.1 (Putnam et al. 2016) in CHiCago mode using the default parameters with the CHiCago library as input.

**Table 1.** Programs used in assembly

Purpose	Program	Version
Oxford Nanopore basecalling	Albacore	2.0.2
Illumina read trimming	SeqPrep2	1.1
Illumina read trimming	Trimmomatic	0.33
De novo assembly	Meraculous-2D	2.2.4
Scaffolding	HiRise	2.1.1
Nanopore adapter trimming	Porechop	0.2.3
Gap-filling	PBJelly	15.8.24
Short-read polishing	Pilon	1.22
Duplicate removal	Samtools rmdup	0.18
Assembly validation	BUSCO	5.0.0
*k-mer* based assembly validation	Merqury	1.1
Masking of repetitive elements	RepeatMasker	1.332 4.1.1
Identification of repetitive elements	RepeatScout	1.0.5
Annotation	Augustus	3.2.2
RNAseq mapping	TopHat2	2.1.0
Protein mapping	Exonerate	2.2.0
Mitochondrial genome assembly	Unicycler	0.4.4
Long read mapping	Minimap2	2.7-r659
Illumina mapping	BWA	0.7.12
Mitochondrial genome iterative assembly	MIA	1.0
Mitochondrial assembly	Jalview	2.11.0

We used Porechop v0.2.3 (https://github.com/rrwick/Porechop) to adapter-trim the ONT reads, and then performed gap-filling on the HiRise assembly using the trimmed long reads (~1.6X coverage of the genome) with the tool PBJelly, part of the PB-Suite v15.8.24 (English et al. 2012). We allowed PBJelly to correct only intrascaffold gaps, and used the default minimum of 1 read spanning a gap.

Next, we used the genome improvement tool Pilon v1.22 (Walker et al. 2014) to correct sequence errors in the gaps that had been filled with the high-error ONT data. We aligned the Illumina shotgun data back to the PBJelly genome using bwa mem v0.7.12 (Li 2013) and marked duplicates with Picard toolkit MarkDuplicates tool (Broad Institute; https://broadinstitute.github.io/picard/). We then ran Pilon with the alignment file as the “– frags” input and the PBJelly genome as the genome file. We specified the genome as diploid and used the default setting to fix all types of changes. We ran 2 iterations of this alignment and consensus sequence calling process, and stopped after the second iteration as no improvement in genome quality metrics was observed.

#### Assembly Validation

We assessed the quality of our genome in 2 ways. First, we used the genome assessment tool BUSCO v5.0.0 (Seppy et al. 2019) to evaluate genome completeness based on a set of conserved single-copy orthologous genes. We used the mammalia_odb10 database, which contains 9226 genes, for BUSCO analysis.

Second, we used Merqury v1.1 (Rhie et al. 2020) to compare *k*-mers (*k* = 21) found in the short-read data used to build the assembly and *k*-mers found in the assembly itself in order to calculate the base-level accuracy (QV) and completeness of the genome. With this approach, *k*-mers that are found only in the assembly, and not in the sequencing data, represent errors, whereas *k*-mers that are found in the reads but not in the assembly likely reflect sequence missing from the assembly. We filtered the read-set *k*-mer database to only those occurring a minimum of 11 times to remove *k*-mers likely originating from sequencing errors.

#### Nuclear Genome Annotation

We first created a repeat-masked reference genome for mapping RNA data. We identified repeat family sequences based on high-frequency repetitive kmers with RepeatScout v1.0.5 (Price et al. 2005) and masked repeats using RepeatMasker v1.332 (Smit et al. 2013–2015). The annotation prediction program Augustus (Stanke et al. 2008) incorporates evidence from different sources (RNASeq, ESTs, proteins) in the form of hints. We converted our multiple evidence formats into hints for input into Augustus in the following manner. We adapter-trimmed short-read Illumina RNAseq data using SeqPrep2 (https://github.com/jeizenga/SeqPrep2) with a quality score cutoff of 15 (-q 15) and minimum read length of reads to 30 bp (-L 30). We aligned these reads to the repeat-masked genome using TopHat2 v2.1.0 (Kim et al. 2013), and filtered the alignment file using bam2hints from the Augustus toolkit v3.2.2 (Stanke et al. 2008), using only intronic regions.We adapter-trimmed ONT RNA reads using Porechop v0.2.3 and mapped these to the repeat-masked genome using gmap v2018-07-04 (Wu and Watanabe 2005), keeping only alignments with maximal scores. We converted the ONT alignments into hints using blat2hints.psl from the Augustus toolkit v3.2.2 (Stanke et al. 2008). We downloaded publicly available proteins from the polar bear (Liu et al. 2014) from gigadb and mapped these to the repeat-masked Andean bear genome using Exonerate v2.2.0 (Slater and Birney 2005), and converted the mapped proteins into hints using exonerate2hints.pl from the Augustus toolkit v3.2.2 (Stanke et al. 2008).

We performed evidence-guided annotation of the Andean bear genome using Augustus (Stanke et al. 2008). We used “human” as the species and allowed for alternatives from evidence (described above), hinted splice sites, untranslated regions, excluded in-frame stop codons, and set the gene model to complete. We converted the output gff3 file into amino acid sequences using getAnnoFasta.pl from the Augustus toolkit v3.2.2 (Stanke et al. 2008) ran the amino acid sequences through blastp (Altschul et al. 1990) to identify them, using the Swiss-Prot database downloaded in May 2019. We filtered hits such that only the top 3 were returned and required that all hits have an e-value cutoff greater than 0.00001. We retained the hit with the highest bit score for each predicted protein. We used the UniProt website to convert the Swiss-Prot IDs of the hits into gene names and GO terms. To evaluate our annotated gene set, we used BUSCO v5.0.0 in protein mode (-m protein) with the mammalia_odb10 database. We also used RepeatMasker v4.1.1 to characterize the repetitive content in the assembly. RepeatMasker was run in sensitive mode (-s) using the Dfam v3.2 database identifying carnivore-specific repeats (-species carnivora).

#### Mitochondrial Genome Assembly

We used Unicycler v0.4.4 (Wick et al. 2017) in hybrid assembly mode to assemble a mitochondrial genome. We reduced our input shotgun data set to only mitochondrial reads by mapping adapter-trimmed Illumina and ONT reads to a previously published mitochondrial sequence for a Andean bear (FM177764.1) (Krause et al. 2008). We used bwa mem (Li 2013) for short reads and Minimap2 v2.7-r659 (Li 2018) for long reads. We converted reads that mapped to the Andean bear mitochondria into fastq format using bedtools bamToFastq (Quinlan and Hall 2010). We used the mitochondrial mapped fastq reads as input into Unicycler, flagging long reads using “l” and unpaired reads using “-.” In order to derive a final consensus from this initial assembly, we used the Unicycler assembly as the reference sequence to perform an iterative reference-guided assembly using MIA (Green et al. 2008; https://github.com/mpieva/mapping-iterative-assembler) with roughly 24 million randomly selected adapter-trimmed Illumina shotgun reads (MIA flags: -i -c -C -U -F -k 13). We noticed a slight reduction in coverage in hypervariable region 2 (HVR2), a region known to have a variable number of repeats. We assessed this region by eye for inaccuracies in the alignment, and manually removed one 17-bp repeat using Jalview (Waterhouse et al. 2009). We repeated the mia assembly with the curated mitochondrial sequence as the reference. We observed no changes in sequence in the outputted mitochondrial assembly, and no longer observed a reduction in coverage across HVR2. Finally, we used MITOS (Bernt et al. 2013) to annotate our final mitochondrial assembly.

## Results

### Sequencing

We prepared 5 different types of sequencing libraries from fibroblast cells, creating an Illumina shotgun library for constructing an initial assembly, CHiCago library for scaffolding, Oxford Nanopore shotgun library for closing gaps in the assembly, and Illumina and ONT RNAseq libraries. We obtained ~1.72B shotgun Illumina reads, which resulted in ~64X coverage used in the assembly after adapter and quality trimming, and ~357M CHiCago reads for use in scaffolding. We also generated 551K shotgun ONT reads, totalling 2.29 Gb (~1× coverage) with a read N50 of 8.4Kb (Supplementary Figure 1). For annotation, we generated 264M Illumina and 32K ONT RNAseq reads.

#### Nuclear Genome Assembly

With an initial Meraculous-2D assembly, our assembly had a total length of 2.08 Gb across 180 766 contigs (139 421 scaffolds) and a contig N50 of 20.1 kb (scaffold N50 of 26.2kb). After scaffolding this assembly with HiRise using the CHiCago library, the scaffold N50 increased to 20.92 Mb (12 402 scaffolds), with a total of 171,229 gaps. HiRise also slightly improved the contig N50, from 20.1kb to 20.3kb. While this was an improvement in contiguity, the short read length of Illumina reads still resulted in a fragmented assembly at the contig level. We, therefore, gap-filled the assembly using long ONT reads. This step closed 8,502 gaps, reducing the number of gaps in the genome from 171 229 to 162 727, and increasing the contig N50 from 20.3 to 21.8 kb and scaffold N50 from 20.92 to 21.12 Mb. We then realigned the Illumina data to the gap-filled assembly to polish the genome in 2 rounds of iterative polishing, resulting in a final assembly of length 2.23 Gb across 12 402 scaffolds (163 790 contigs) and a scaffold N50 of 21.12 Mb and contig N50 of 23.5 kb. Scaffolds larger than 1Mb constitute roughly 85% of the genome.

#### Assembly Validation

Standard metrics of assembly completeness and coverage after each step in the assembly process are provided in Table 2. We used 2 approaches to validate the completeness and accuracy of our final assembly. Our final assembly has a BUSCO score of 88% ([S:87.4%, D:0.6%], F:4.0%, M:8.0%; n:9226) complete single copy orthologs using the mammalia_odb10 database. Using Merqury, we obtained *k*-mer-based estimates of error and completeness in the assembly. The QV score for the assembly, which reflects the Phred-scaled error rate, was 34.36, reflecting an estimated error rate of 0.00037. The assembly completeness was 93.07%. However, *k*-mers in the read set originate from both haplotypes, so a portion of those absent in the assembly represent missing heterozygous variants (Supplementary Figure 2).

**Table 2.** Genome metrics of Andean bear assembly stages

		Assembly Version				
		Meraculous-2D	HiRise	PB Jelly	Pilon iteration 1	Pilon iteration 2
Assembly step		Shotgun assembly	Scaffolding	Gap filling	Error correcting	Error correcting
Input data		Illumina shotgun	CHiCago	ONT	Illumina shotgun	Illumina shotgun
Genome length (bp)		2 086 202 895	2 215 996 330	2 232 824 675	2 232 974 266	2 232 736 973
Contigs		180 766	183 631	166 073	163 790	163 790
Scaffolds		139 421	12 402	12 402	12 402	12 402
Contig N50 (kb)		20.1	20.3	21.8	23.2	23.5
Contig L50		31 094	30 649	28 724	27 028	26 657
Scaffold N50 (Mb)		0.0262	20.92	21.12	21.12	21.12
Scaffold L50		23 560	29	29	29	29
Number of gaps		41 345	171 229	162 727	153 671	151 388
Number of Ns		1 438 142	131 322 142	125 625 584	124 345 965	123 688 823
% of Ns in genome		0.00198	5.93	5.62	5.57	5.54
Busco Scores (%; *n* = 9229)	C	54.9	87.9	88.0	88.1	88.0
	S	54.6	87.4	87.4	87.5	87.4
	D	0.3	0.5	0.6	0.6	0.60
	F	16.2	3.9	4.0	3.9	4.0
	M	28.9	8.2	8.0	8.0	8.0

The metrics of the Andean bear genome at different stages of the assembly process. The final column represents the final assembly. We saw a marked improvement in N50 as a result of scaffolding with HiRise. Gap filling with PBJelly noticeably decreased the numbers of Ns and strings of Ns. As a result of this conversion of Ns to useful sequence, the number of Illumina reads that mapped to the assembly increased. Full BUSCO scores are shown at the bottom, indicating the proportion of complete (C), single-copy (S), duplicated (D), fragmented (F), and missing (M) conserved single-copy mammalian orthologs that a present at each stage of the assembly.

#### Nuclear Genome Annotation

**Figure 1.**
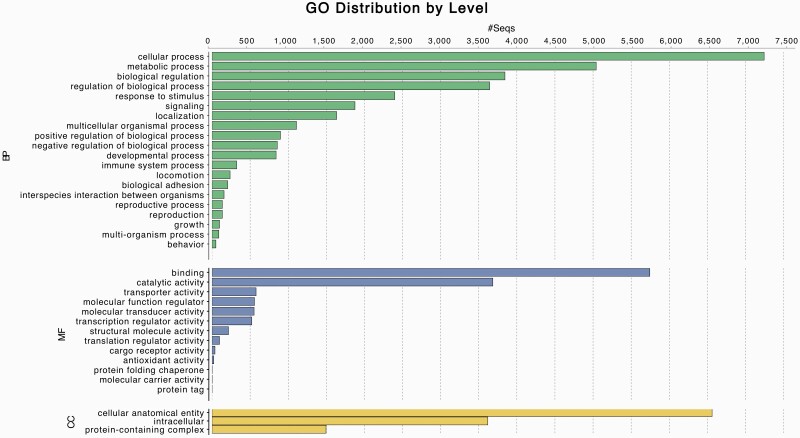
Top 20 Gene Ontology term names in Andean bear annotation. Annotation by Augustus predicted a total of 19 289 genes. After filtering, this translated to 15 504 annotated proteins, which we subclassified into the 3 GO domains: biological process (green), molecular function (blue), and cellular component (yellow), and visualized results in Blast2GO (Götz et al. 2008). The 20 most common GO term names appearing in the annotated gene set are shown.

Annotation by Augustus predicted a total of 19,289 genes. After filtering, this translated to 15,504 annotated proteins, which we subclassified into the 3 GO domains: biological process, molecular function, and cellular component, and visualized results in Blast2GO (Götz et al. 2008) (Figure 1). To assess the quality of the annotated gene set, we ran BUSCO in protein mode, obtaining a BUSCO score of 72.7% ([S:55.0%, D:17.7%], F:8.9%, M:18.4%; n:9226). RepeatMasker identified 30.89% of the genome as repetitive, similar to what has been found for other ursid species (Srivastava et al. 2019).

#### Mitochondrial Genome Assembly

We created an initial mitochondrial genome assembly with Unicycler v0.4.4 using both short and long shotgun reads, and then refined this assembly using short reads with the iterative assembler MIA. Following manual curation to remove one 17 bp repeat from the assembly, the final mitochondrial genome was 16,722 bp in length. We used MITOS to annotate the mitochondrial assembly (Supplementary File 1). This annotation suggested that we had assembled the complete mitochondrial genome.

## Discussion

**Figure 2.**
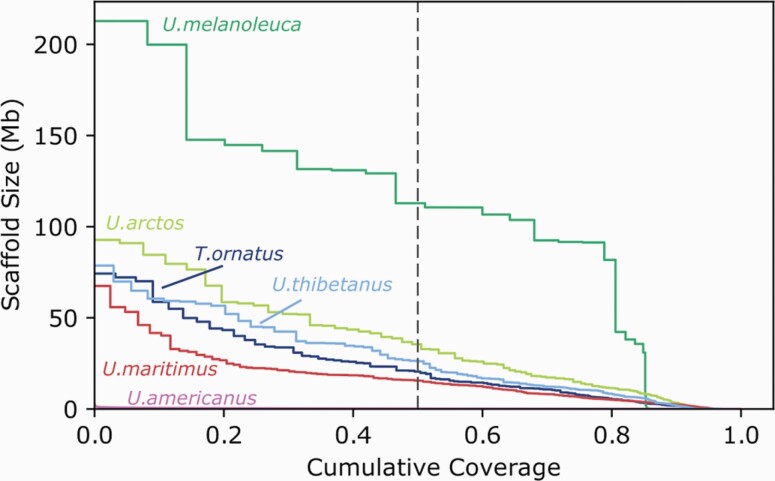
NGx plot comparing scaffold-level contiguity of Andean bear assembly and other bear genomes. Plot shows the fraction of the genome (x-axis) that is covered by scaffolds of at least a certain size (y-axis). The dashed vertical line shows this value for half of the genome (N50). The Andean bear (*T. ornatus*) genome is shown in dark blue, with the giant panda (*Ursus melanoleuca*) in dark green, brown bear (*U. arctos*)in light green, Asiatic black bear (*U. thibetanus*) in light blue, polar bear (*U. maritimus*) in red, and American black bear (*U. americanus*) in pink. The American black bear genome was assembled using short read data alone, whereas all the others were assembled using a combination of short read, long read, and proximity ligation data. The assembly contiguity is similar to that of other the bear reference genomes that also employed a hybrid assembly approach.

The Andean bear genome assembly we present here is highly contiguous and complete, with low error. This assembly shows the value in combining multiple data types, such as short-read, long-read, and proximity-ligation sequencing data to create a high-quality, annotated, draft genome. Although our assembly is more fragmented at the contig level relative to assemblies built primarily with long-read data (Rhie et al. 2021), the approach taken here highlights that it is possible to use low-coverage long-read sequencing to resolve many of the gaps inherent in short-read assemblies. With additional scaffolding via proximity-ligation data and correction using the short-read Illumina data, this results in a draft assembly that is more contiguous than assemblies built using short-read data alone while maintaining the low base error rate of Illumina assemblies (see Figure 2 for a comparison of contiguity with available bear reference genomes, most of which were built using a similar combination data types). We also took a hybrid approach in annotating the genome using RNAseq data, and show that combining short and long reads successfully generates an annotation across a range of gene functions (Figure 1).

This is the first contiguous draft genome assembly from a tremarctine bear and will provide opportunities for comparative genomic studies of bears. With this draft assembly, along with assemblies already available from the other 2 bear subfamilies, Ailuropodinae and Ursinae, including panda, black bear, brown bear, and polar bear, there are now draft genomes from each of the 3 subfamilies in Ursidae. Bears have been a focus of evolutionary research due to their broad geographic distribution and diversity of ecological niches that they inhabit (Kuduk et al. 2012; Miller et al. 2012; Liu et al. 2014; Rinker et al. 2019). The availability of an Andean bear genome allows both greater sensitivity in detecting selection in other lineages and more detailed assessment of the unique adaptations in Andean bears, including high-altitude adaptation (Witt and Huerta-Sánchez 2019) and adaptation to a more herbivorous diet relative to other bears (Peyton 1980). The genome will also help to resolve the relationships between Andean bears and the 2 recently extinct tremarctine bear lineages, *Arctodus* and *Arctotherium* (Matheus 1995; Soibelzon et al. 2005; Mitchell et al. 2016). A draft Andean bear genome will also improve analyses of the complex history of admixture among bear lineages in the subfamily Ursinae (Kumar et al. 2017,Cahill et al. 2015, 2018) that have largely relied on the panda, which is ~20 million years diverged from ursine bears (Hu et al. 2017), as the closest available outgroup for use in detecting admixture, compared to an ~11 million year divergence between tremarctine and ursine bears (Kumar et al. 2017).

Finally, the Andean bear genome is a useful tool to augment conservation efforts of the species, which is classified as “Vulnerable” by the IUCN (Velez-Liendo X & García-Rangel 2016). Habitat fragmentation from mining and oil exploitation have left Andean bear populations in isolated segments of intact habitat scattered throughout the overall range (Kattan et al. 2004). Whole-genome information will provide much finer resolution insight into connectivity between patches and inform on the overall genetic diversity of the species, as compared to approaches that use limited numbers of markers, such as microsatellites. Small, isolated populations are more subject to genetic drift and inbreeding (Saccheri et al. 1998; Saremi et al. 2019), and so habitat fragmentation may result in the loss of Andean bear genetic diversity overall (Cueva et al. 2018). In addition, because, evaluating population viability of the fragmented, low-density population of Andean bears poses logistic challenges in sample collection and disturbance of wild populations, the use of fecal or environmental DNA has been recommended for population genetic assessments of inbreeding, dispersal, and gene flow. A draft reference genome facilitates the development of robust tools for population assessments, monitoring, and potential management interventions (Viteri and Waits 2009).

## Supplementary Material

esab021_suppl_Supplementary_Material

## Data Availability

We have deposited the project in NCBI under the project number PRJNA472085. Sequence data used in the assembly is available under the IDs SRR13602715-SRR13602719. The genome is available in Genbank under the accession WMLG00000000. The Andean bear mitochondrial genome is available on Genbank under the accession number MW556430.
