# Supplementary material for: An Annotated Draft Genome for the Andean Bear, Tremarctos ornatus
Source: J Hered. 2021 Apr 21;112(4):377–84. doi: 10.1093/jhered/esab021 (PMC8280923; doi:10.1093/jhered/esab021)
Supplement: esab021_suppl_Supplementary_Material [file esab021_suppl_supplementary_material.docx]

**Supplementary Material**


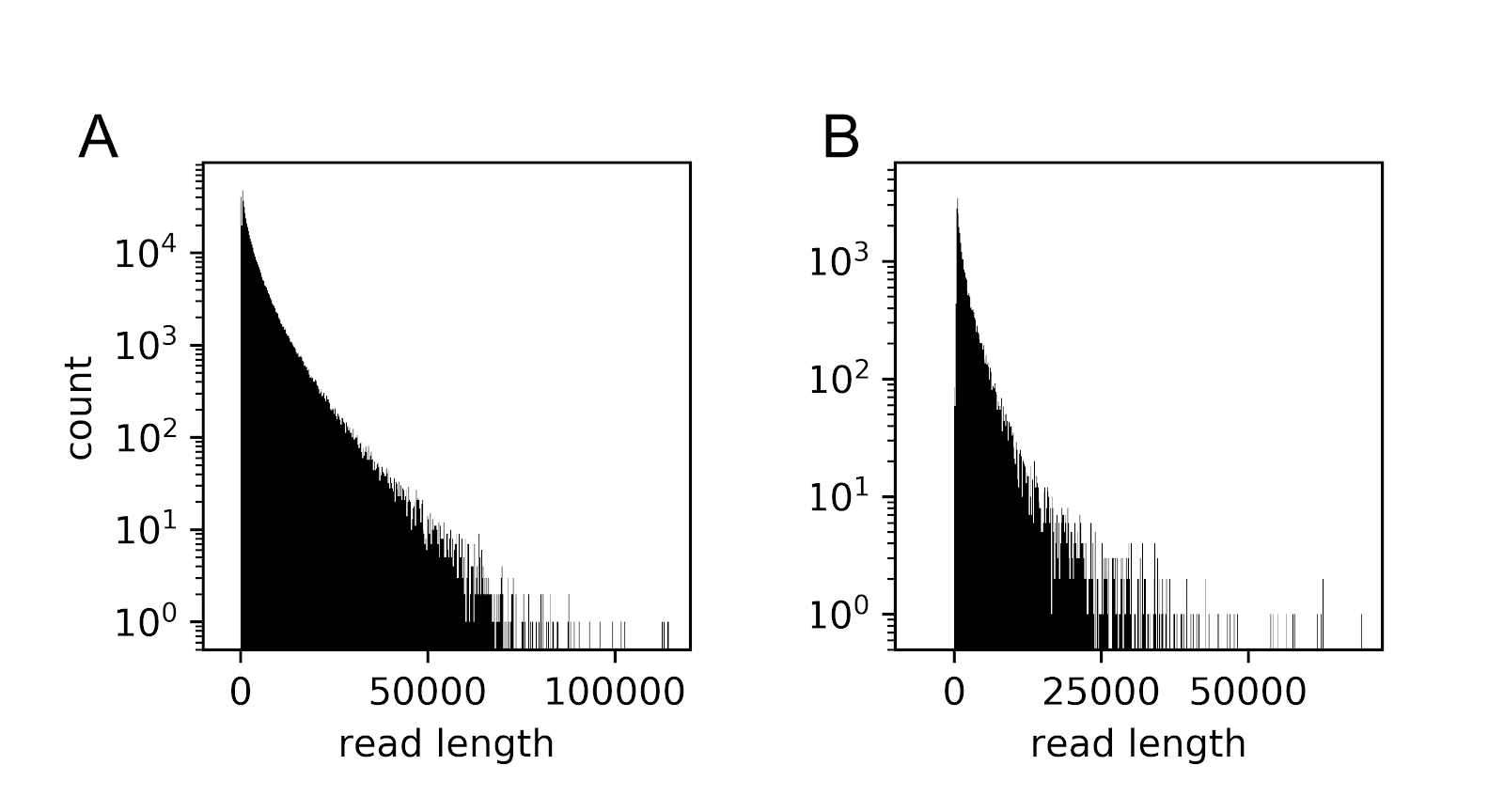


**Supplementary Figure 1.** Histogram of read lengths for the shotgun (A) and RNAseq (B) Oxford Nanopore reads used in the assembly.


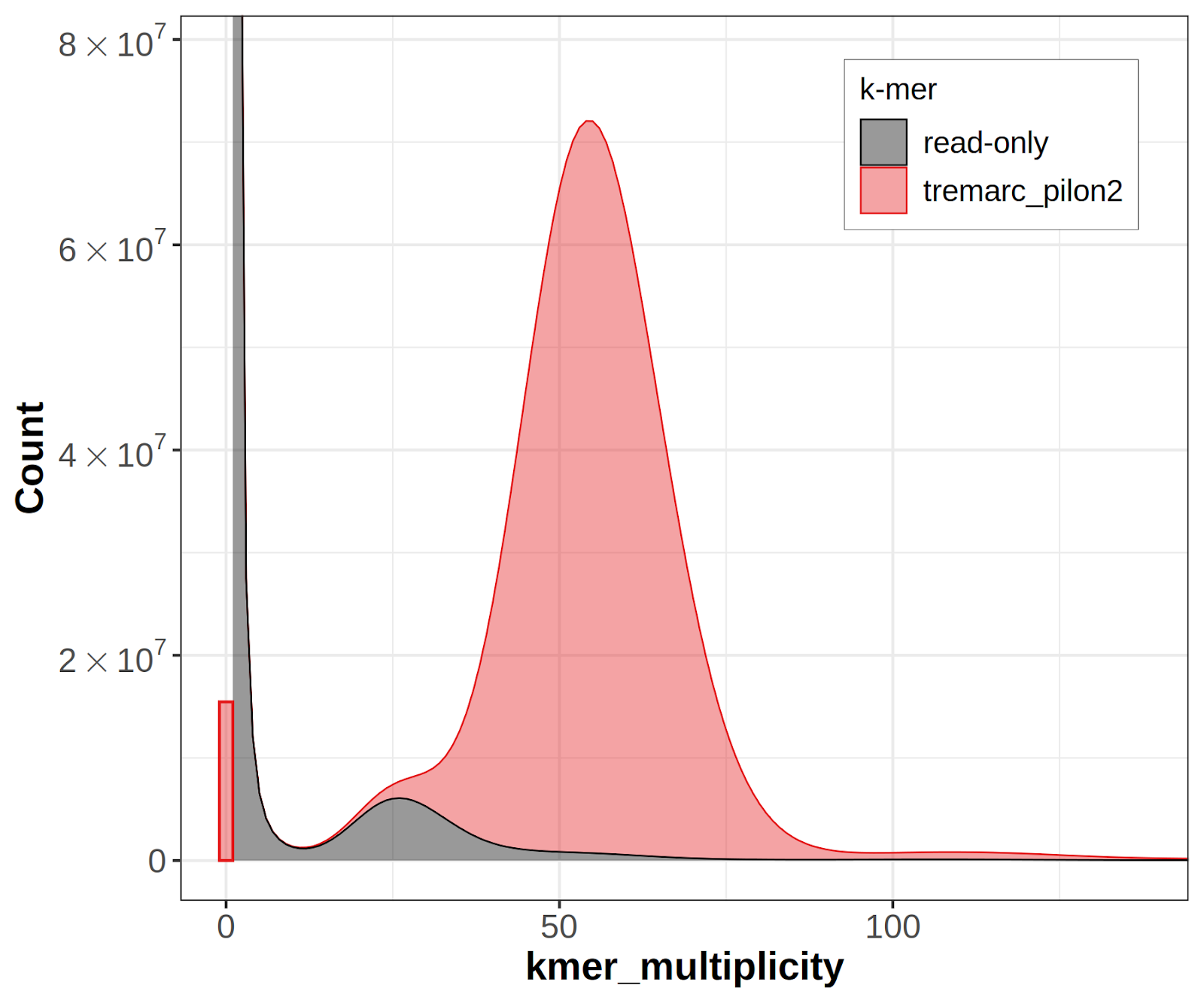


**Supplementary Figure 2.** Spectra-asm plot comparing the *k*-mer content of the final assembly and the Illumina read set used in the assembly. *K*-mers found only in the read set are depicted in gray. Those found in both the assembly and the reads are shown in red with a multiplicity of 1 or greater. The *k*-mers found only in the assembly are those shown in red with a multiplicity of 0.

**Supplementary File 1**. BED file containing annotation of Andean bear mitochondrial genome.
